# Non-application of the nursing process at a hospital in Accra, Ghana: lessons from descriptive research

**DOI:** 10.1186/s12912-018-0315-x

**Published:** 2018-11-13

**Authors:** Joana Agyeman-Yeboah, Kwadwo Ameyaw Korsah

**Affiliations:** 1 International Maritime hospital, Tema, Ghana; 2 School of Nursing, University of Ghana, P. Box LG43, Legon, Ghana

**Keywords:** Nursing process, Nursing care-plan, Non-usage, Nurses

## Abstract

**Background:**

Registered nurses in Ghana are trained to plan the care that they provide to their patients in a systematic and organized manner. This scientific approach to care is known as the nursing process. There is evidence that the nursing process is not being practised by professional nurses in Ghana, as expected. This research seeks to explore what informs nursing interventions in the clinical area.

**Methods:**

A qualitative study was conducted with ten registered nurses; and this was descriptive in nature. One-on-one interviews were conducted with the research participants, as a means of collecting the data. A semi-structured interview guide was used as the data-collecting tool. The collected data were analysed by using latent-content analysis. Three main themes emerged from the data analysis.

**Results:**

It was found that registered nurses did not plan their nursing care. The care that the nurses provided was based on routine nursing care and doctors’ orders, both verbal and non-verbal; or written communication were the means whereby the care was provided; and that was communicated among the nurses.

**Conclusion:**

Registered nurses are taught the nursing process; and they are expected to implement the acquired knowledge in the clinical area. The failure of nurses to practise the expected standard of care results in their relying on the decision of other health-care professionals, such as doctors. This makes registered nurses appear to be assistants to doctors. We, therefore, conclude that nurse leaders must supervise nurses to put into practice what they were taught during their training; so that they can have professional autonomy in their practice as nurses. It is also suggested that nurses must show evidence of using the nursing process in their daily work by the use of the nursing care-plan form.

**Electronic supplementary material:**

The online version of this article (10.1186/s12912-018-0315-x) contains supplementary material, which is available to authorized users.

## Background

Registered nurses are expected to be able to identify the client’s health problems, with the intention of preventing, protecting and promoting the wellbeing of the patient. Whatever help the patient may require, in order for his or her needs to be met, be it physical, social, economic, or psychological; while he or she is undergoing some form of medical or surgical treatment, it is the nurse’s responsibility to see to it that these needs are met – either directly by his or her own activity, or indirectly by calling in the help of other healthcare professionals [1]. The nursing process provides a framework that can be used in all nursing situations [2].

It has been defined as an orderly, systematic manner of determining the client’s health status, specifying those problems, which have been defined as alterations in human needs; and making plans to solve them – and implementing the plan, as well as evaluating the extent to which the plan was effective in promoting optimum wellness and resolving the problems identified [3].

According to Carpenito-Moyet [4], “The nursing process and [the] nursing diagnoses are often a visible subject in each course syllabus; but in reality, classroom teachings and discussions are focused predominantly on medical diagnoses. Nursing students are therefore guided by their tutors to learn how to make medical diagnoses; and they are left on their own to learn nursing diagnosis. Thus, in the end, medical diagnoses guide their practices, leaving nursing diagnoses as only an unpleasant memory. Often, the nursing tutors teaching the nursing process do not understand nursing diagnoses; and therefore, do not embrace, value, or teach them. A lot of students spend hours creating care plans by copying from textbooks, without learning the critical thinking skills needed for the analysis of the data they have obtained; hence, the nursing diagnoses have no relevance to them; and they remain irrelevant to them after graduation” (P. 126). It is evident that nurses find it difficult to implement the nursing process; because some professional nurses do not quite understand it [5].

A study conducted in Ghana on factors that influence clinical utilization of the nursing process found that, registered nurses were not formulating, or stating the nursing diagnosis. They carried out various nursing interventions that were not based on the nursing diagnosis. They were also not evaluating the rendered care; thus the nursing process in totality was not being implemented [6]. If professional nurses in Ghana are not implementing the nursing process, what then informs their practice? The purpose of this study was to explore and describe the extent to which registered nurses working at the 37th Military Hospital implement the nursing process in the clinical area.

## Methods

The study aimed to explore what informs nursing interventions in the clinical area. A qualitative research design was employed, which was descriptive in nature. This design gave the participants the opportunity to express their opinion on the situation being studied. The study was conducted at the 37 Military Hospital. This is a 400-bed hospital located in Accra, the capital of Ghana. The participants consisted of five nursing officers, two senior staff nurses and three staff nurses. Their ages ranged from 27 years to 41 years; and the range of the years that they have been working was from 4 years to 9 years. The male-to-female ratio of the participants was 1:9.

The participants were selected by using the purposive-sampling technique. Arrangements were made with those who expressed interest in the research. The researcher met them; and they were briefed about the research. They were informed about the purpose of the study; and how the data would be collected from them. They were assured of their confidentiality. How the data would be managed was also explained to them. They were informed that their participation in the research would not affect their employment in the hospital. Their right and freedom to withdraw from the study, at any time, was explained to them. The research participants were also given the opportunity to ask any questions to clarify any issues related to the study.

Those willing to participate in the study were given the consent form to read; and further explanation was given on what was stated in the form. Those who accepted to participate in the study were allowed to sign the consent form. They were given a copy of the signed consent form to keep. An arrangement was made with the participant for a convenient time and place for the interview.

A semi-structured interview guide was used as the data-collecting tool (attached as an Additional file 1) This interview guide was first piloted in a public hospital in Accra, in order to test the validity of the tool. After the pilot study, the interview guide was slightly amended.

The participants were interviewed until the tenth participant, after which no new information was forthcoming. The data analysis was done concurrently with the data collection. The researchers observed from the data analysis that no new themes or concepts were emerging from the interview; and therefore, the researchers stopped the interviews. The interviews were recorded on a voice recorder with the permission of the participants; and points on key issues were also written down as part of the field notes during the interview. Each interview lasted for approximately forty-five minutes to one hour. The data in this study were analysed by using latent-content analysis [7].

Immediately after the interview session, the recorded interviews were replayed and transcribed verbatim. The data were then coded; and during the coding, the data were read by the researcher several times. Sections of the text were highlighted; and comments were made regarding anything that was striking. These comments included overall impressions; and points of interest were written in a margin created at the right side of the sheets. After coding, the data were organised with related codes; and they were put into categories [7]. The coding and analysis of the data was done by an independent coder.

Consensus was reached between the researchers and the independent coder, before the themes were finalized. A label was given to each category. Once the data were categorized, a summary for each of the categories was written. How the categories relate to each other was carefully analysed; and then the themes were developed.

## Results

Three main themes emerged from the data analysis. These were: nursing care is not planned by nurses; alternatives to the nursing care plan and means of communicating nursing care. Two of the main themes had their respective sub-themes. These are described below:

### Nursing care is not planned by nurses

Unplanned nursing care was a major theme that emerged from the analysis. By this it is meant that nursing care is provided to the patient, without any formal care plan as a guide. Nurses stated they knew they must plan care; but they did not do it. Instead, they provided nursing care, based on what they perceived the patient needed at that time. Some nurses stated that this lack of formal plan of care was due to the high patient–nurse ratio and the inadequate time available. This was expressed by one participant as follows:

*“We actually do not have a plan per se, when we admit a patient, we do not plan the care of the patient right on admission. I think what we do is that from time-to-time, we meet the needs of the patient, as time goes on, or when the need arises; but ideally, we are supposed to plan the patient care, according to the diagnosis and treatment of the patient.”*

Another participant also felt that they were not able to plan the care of the patient; because they needed to care for a lot of patients at a particular time; hence, they do not get the necessary time to plan the care; although they are aware that planning of the care is the correct thing to do. One of them stated it in this way:

*“Ideally, we are supposed to use the nursing-care plan; but because of the low nurse-to-patient ratio, we do not get the time to do that. We usually care for the patient and what their needs are immediately; but we do not use the nursing process.”*

It was evident from the data analysis that, when a patient comes for admission to the ward, the nurses do not plan the care of that patient with the patient. Instead, just as they decide the overall care for the patient, they again use their discretion. One participant indicated it this way:

*“As for the planning, it is not done; it is just that when the patient comes on admission, we use the admission books like the nurses’ notes, the temperature, pulse and respiration chart; and then we follow the doctor’s orders from the folder; and we also use our discretion to nurse the patient.”*

Although no planning is made in relation to the care that the patient receives; some of the participants did not really see this as a problem. They indicated that it was a nation-wide situation; and that it was not peculiar to the institution in which they work. One of the participants expressed his view by stating that:

*“I will say even in this hospital, I don’t know whether we even plan the care. Planning is not done; we really care for the patient, but not with the care plan. I think the problem is not just with the 37 Military Hospital; but I think it is a nation-wide problem. The nursing process in actual fact is not being used well in Ghana. I think it is a foreign concept; and also the high patient to nurse ratio is a contributing factor.”*

The above statement from the participants reveals that they are aware of the existence of the nursing-care plan. They are also aware that they are supposed to plan the care of the patient; but they are not doing it.

### Alternatives to the nursing-care plan

This theme describes what nurses actually depend on to care for the patient instead of developing and using the care plan. Two sub-themes emerged: (a) care based on nursing routines; and (b) care based on the doctor’s orders.

#### Care based on nursing routine

Some of the participants indicated that the care that they provide to the patient is based on the daily routine care that they normally provide for all the patients, regardless of their medical diagnosis. This was how a participant stated it:

*“We do the normal routine nursing care, like checking the temperature, blood pressure and serving of medication; and if the patient needs any more care, the patient would call; and we would then attend further to the patient.”*

From the experiences that the nurses had, they knew what was normally done for the patients during the morning, afternoon or the night shift; hence when the nurses come to work, they go strictly according to the daily routines. For patients who are able to verbalize their problems and request particular care; the nurses would attend to these requests and try to meet their needs. One of the participants had this to say:

*“In the morning we take over from the night staff by moving from bed to bed; and if along the line there is any special plan about a specific client that we need to be notified, the night staff do that. If we need to bring that to the attention of the doctor, we do that; and we also do the bed-making; we tidy up the ward; and we then carry on with the other daily routines, such as the dusting of the ward, as well as the nurses’ station.”*

Another participant with a similar view stated:

*“Depending on the shift in which you are coming; if it is in the morning shift, you know you have to prepare the patient for the ward rounds, feed those who cannot feed themselves, serve medications; and sometimes even sit by the patients and converse with them. If you come for afternoon shift too, the routines are the serving of medications, talking with the patients, turning those who are bed-ridden, or feeding them when necessary.”*

The nurses were aware of what they were expected to do for the patient, as a routine activity, depending on the shift in which they are working. These routine activities were not formally planned, nor based on the specific needs of the patient; but they were carried out as a daily formality, irrespective of the patient’s condition. Patients who were able to verbalize their problems receive the necessary care to assist them in solving their problem.

#### Nursing care based on doctors’ orders

The participants indicated that they depend on the orders or the instructions given by the doctor, as stated in the patient’s folder, in order to care for the patients. They follow these instructions to provide the care of the patient, instead of, as one participant noted, critically thinking about the presenting complaints of the patient and then planning care that will be holistic and individualized, depending on the health needs of the patient. This tends to make the nurses dependent on the doctors. A participant passionately expressed this as follows:

*“I have also observed that our seniors see themselves as subordinate to the doctors; so they tend to follow whatever the doctors say, instead of them actually thinking critically, to be able to analyse things and to do what is right for the patients. This, therefore, does not actually cause the nurses to think, and then to use all that they were taught in school.”*

The participants pay particular attention to what the doctors write in the patient’s folder; and they then follow those instructions strictly. This was how a participant expressed it:

*“When a patient comes to be admitted, after the admission, I think; whatever the medical instructions, which have been given or the medical orders, are implemented.”*

Although the nurses followed strictly what the doctors have written in the patient’s folder, one of the participants saw the practice of taking orders from the doctors and not planning the care by coming out with nursing orders to guide the nursing care, as not being good for the nursing profession. He believed that nurses are expected to have their own nursing orders, as well as implementing the medical orders. He stated:

*“That is why I am saying that the nursing we are practising now is more dependent on medical orders than nursing orders, which is not always too good for the profession.”*

Adherence to the daily routines usually performed by the nurses and the implementation of medical orders stated by the doctors in the folder were the two main alternate means, which the participants stated that the nurses uses instead of the nursing care plan. There were some perspectives put forward that nursing care could have been more client specific; and furthermore, it could focus more specifically on addressing the identified health problems; so as to meet the needs of the patient holistically.

### Means of communicating nursing care

This theme describes the various means by which nurses communicate the care provided to the patient with one another, as well as how they document what is yet to be done for the patients. Two sub-themes emerged from this theme; and they were: (a) verbal communication and (b) non-verbal, or written communication.

#### Verbal communication

The participants indicated that they do not plan the care of the patient, but nursing interventions that have already been carried and are yet to be implemented, they communicate verbally with each other. This occurs during the handing over of care from one shift to the next. At change of shift, they verbally tell the incoming nurses what has been done for the patients. One of the participants explained this as:

*“During the handing over, communication of the rendered care is done orally, but the in-coming nurses also read through the notes written in the changes book, before we start the handing over. The out-going nurses will tell the in-coming nurses whatever care that has been rendered to the patients in their absence.”*

Another participant also indicated that:

*“We do that orally; as we change over from one shift to the other. All reviews made by the doctors are documented in the changes book. We hand it over orally to the in-coming nurses; but it is also recorded in the changes book, as well.”*

The verbal communication was not only used to throw more light on what has already been done for the patient and documented. It was also used to tell the incoming nurse of things that are supposed to be done for the patient. This was evident in the comments of one of the participants, who developed a strategy to note the verbal information down, in order for him not to forget:

*“I have a notebook; in which I document all the information that is handed over to me. If I am not able to attend to the needs of all the patients; then I hand over what could not be performed to the in-coming nurse. But it is not documented in the care plan, which would have been easier for the incoming nurse to know what has already been done and what is yet to be done, which would have enhanced the continuity of care.”*

Verbal communication was used by the nurses to communicate documented and undocumented nursing care; but this was not done with the use of the nursing care plan.

### Non-verbal or written communication

It was expressed by the participants that all the nursing care provided by the nurses is supposed to be documented in the nurses’ notes, the changes book, and the 24-h report book. These are expected to be read by the incoming nurses, to enable them have an idea of what has already been done for the patient in the previous shifts; and also to be informed about what they are supposed to do for the patients, and at what time. One of the participants had this to say:

*“The care provided is written in the changes book for the in-coming nurse to read. During the handing over, the nurse is told what has already been done, and what is yet to be done.”*

Another participant also expressed it as follows:

*“We write every procedure we do for the patient in the nurses notes and in the changes book; so when new nurses come or somebody comes in the afternoon to take over from me, the person reads the changes book and the nurses’ notes at the nurses’ station, goes through everything to see what has been done for the patients in his or her absence, before going round to say hello to the patients.”*

One other participant stated that:

*“We usually write notes on patients in the changes book and in the nurses’ notes. This informs the incoming nurse of what has been done for the patient and the things that are pending or yet to be done for the patient.”*

The care provided by the nurses is documented in the nurses’ notes, the changes book and the 24-h report book. The care that is yet to be provided is at times not documented; but verbally handed over. If the nurses were using the nursing care plan, the rendered care would have easily been documented on the care plan; and what was yet to be carried out would have been stated under the nursing orders, which would have made the handing over procedure easy; and this would also have ensured continuity of care.

## Discussion

The purpose of the study was to explore what informs nursing interventions in the clinical area; since the nurses were not using the nursing process. The participants comprised 9 females and 1 male. This outcome was not surprising, because of the highly dense population of females in the nursing profession in Ghana than males. However, this observation was in sharp contrast to that made by Auerbach, Buerhaus, Staiger and Skinner [8]. The findings from this current study revealed that, the nurses in the study stated they knew they must use the plan care; but they did not do so. In addition, they did not plan the care of the patients; instead, they provided nursing care based on what they perceived the patient needed at that time; and they decided the overall care for the patient by using their own discretion. The perception of the nursing process as a foreign concept might have also affected their non-utilization thereof.

Unlike the findings from an earlier study by Hayrinen, Lammintakaren and Saranto [9], the planned or performed nursing interventions were documented by using the nursing-process model. A systematic review on nursing diagnosis, interventions and outcome, application and input on nursing practice reported that nursing diagnoses are commonly found in nursing practice [10]. What Hayrinen, Lammintakaren and Saranto [9] and Muller-Staub, Lavin and Needham [10] are saying seems to be pointing at a common issue regarding the documented use of the nursing process in patient care, which is generally accepted as a scientific way of planning and implementing patient and client care by professional nurses [11].

This is in sharp contrast from the findings of the current study, which has noted that the nurses provided nursing care to patients, based on their own discretion or preference that appears to be intuitive or instinctive in nature grounded on knowledge and experiences in practice by the nurses. This may be seen as an insentient or unconscious awareness of reasoning and cognition [12]. As researchers who have also practised as professional nurses for over 17 years, we have experienced this phenomenon of intuitive reasoning among nurses during patient care in selected hospitals in Ghana, in which we have worked.

The study also revealed that nurses normally provided daily routine care for all the patients, irrespective of their medical diagnosis. These routine activities were not formally plan-based on the specific needs of the patients; and those patients who were able to verbalize their problem received care to assist in solving their problem. The participants’ care that they provided to their patients was based on the medical orders, or the instructions stated in the patient’s folder by their doctors. This assertion is similar to the findings of Carpenito-Moyet [4], who reported that nurses are perceived as assistants to the doctors; but not as professionals in their own right.

The nurses in this context exclusively focused on the problems of the patients that are associated with medical diagnosis or treatment; hence, failing to embrace professional nursing. From the researchers’ clinical experiences, nurses follow the medical orders of doctors to the last; and at times, they are unable to provide any better care to patients who in a specific day were not reviewed by their medical doctors. Hence, the nurses do not take the initiative in patients’ care; as professionals are expected to do.

The findings from this study also revealed that the participants documented the nursing care provided to the patients in the nurses’ notes, the changes book and the 24-h report book, but not in the nursing care plans. Since there is no plan on the care, there is no documentation on the next care to be received by the patients. Verbally, these unplanned cares are handed over to the next shift. What then happens if the outgoing nurse forgets to inform the incoming nurse of this undocumented care? Implementation of the nursing process and the dutiful use of the nursing care plan is therefore very crucial in professional nursing practice; and it should not be trivialized by nurses.

### Limitations of the study

The study was conducted in only one Hospital; and consequently the findings could be specific to only that hospital. The findings from this current study could also not be generalized, due to the qualitative nature of the research design.

### Recommendations

#### Based on the findings, we recommend the following

- Nursing administration should put measures in place to ensure that the nurses are well supervised, in order to practise what they have been taught from school. For instance, the principles underlying “nursing audit” may be applied in hospitals, in which nursing administrators may be tasked to visit hospital wards on a regular basis, in an effort to appraise the patient care rendered by nurses with particular reference to the application of the nursing process among other nursing interventions. Nursing audit is a thorough evaluation and assessment of designated or particular clinical accounts by qualified professional nurses; or it is an assigned duty for the personnel to appraise the value and worth of the nursing care rendered to the patients/clients [13].
- During the handing over of the care rendered to the patients by the nurses, the care plan should be a means of communication and it should be part of the patient’s folder. Since the care plan would have the client’s health problem, the nursing diagnosis, the outcome criteria, the nursing orders and the nursing interventions documented, the incoming nurse at a glance of the care plan will have an idea of the interventions that have already been carried; out and what is yet to be carried out. Additionally, the extent to which the objectives set to address the clients’ problem have been achieved would also be documented in the care plan. This would also help to avoid any repetition of the care that did not assist in meeting the outcome criteria of the patient.
- Nurses must show evidence of using the nursing process, in order to qualify them for promotion to the next level in their career. If this is enforced, nurses would be compelled to practise what they were taught in nursing school; and this would also contribute to the promotion of standards in the nursing profession.
- Nursing lectures should receive in-depth training on strategies to transfer the knowledge on the concept of the nursing process to student nurses in such a way that implementation of the concept after school does not just become a challenge.
- There should be further studies done in other parts of the country on the non-utilization of the nursing process to ascertain the greater picture of the problem in the country as a whole.

## Conclusions

Professional nurses in Ghana’s non-usage of the nursing process is worrying; since they are not implementing theoretical knowledge in the clinical setting. The failure of nurses to practise the expected standard of care results in them relying on the decision of other health-care professionals, such as the doctor, thereby making nurses appear as mere assistants to doctors. We therefore conclude that nurse leaders must supervise nurses to put into practice what they were taught during their professional training; so that they can have professional autonomy in their practice as nurses.

## Additional file

Additional file 1: Interview guide. (DOCX 13 kb)

## Abbreviations

IRB: Institutional Review Board
