# Supplementary material for: Non-application of the nursing process at a hospital in Accra, Ghana: lessons from descriptive research
Source: BMC Nurs. 2018 Nov 13;17:45. doi: 10.1186/s12912-018-0315-x (PMC6234559; doi:10.1186/s12912-018-0315-x)
Supplement: Supplementary file 1 — Interview guide. (DOCX 13 kb) [file 12912_2018_315_MOESM1_ESM.docx]

**Interview Guide**

**Section A**

Socio-demographic data

1. Age
2. Sex
3. Ward
4. Rank
5. For how long have you been practicing as a nurse?
6. How long have you been working on your current ward?

**Guiding Questions**

**Section B (Views of Nurses on the use of the Nursing Process)**

1. How do you plan your care for a patient (E.g. newly admitted or already admitted)?
2. How do you communicate your care to your colleagues?
3. Tell me about what you use to plan the care of patients apart from the nursing process?
4. What else do you want to share with me on the use of the nursing process?
